# Maternal Attitudes about Objectively Monitored Bednet Use in Rural Uganda

**DOI:** 10.1155/2016/8727131

**Published:** 2016-10-19

**Authors:** Paul J. Krezanoski, Data Santorino, Nuriat Nambogo, Jeffrey I. Campbell, David R. Bangsberg

**Affiliations:** ^1^Department of Medicine, Massachusetts General Hospital, Boston, MA, USA; ^2^Department of Pediatrics, Massachusetts General Hospital, Boston, MA, USA; ^3^Harvard Medical School, Boston, MA, USA; ^4^Mbarara University of Science and Technology, Mbarara, Uganda; ^5^Consortium for Affordable Medical Technologies (CAMTech), Mbarara, Uganda; ^6^Oregon Health Sciences University and Portland State University School of Public Health, Portland, OR, USA

## Abstract

Insecticide-treated bednets (ITNs) are a mainstay of malaria prevention, yet poor adherence poses a major barrier to effective prevention. Self-reports of bednet use suffer from recall and social desirability biases. We have designed a device that electronically records ITN usage longitudinally. SmartNet consists of circuits made from a conductive fabric interwoven into the sides and top of a rectangular ITN. Digital sampling of the state of these circuits allows for determining whether the SmartNet is deployed for use or folded up. We conducted a study among pregnant women and women with children <5 years in Uganda to determine attitudes about objective bednet monitoring and SmartNet. Fifty women were interviewed with an average age of 27 years and 2.3 children. Twenty-two percent were pregnant. Ninety-five percent had used a bednet and 90% reported having a bednet at home. After displaying a SmartNet, 92% thought it would be easy to use and 100% expressed interest in using SmartNet. Concerns about SmartNet included washing the net, worries about being monitored while asleep, and worries about users removing the device components. Objective monitoring of ITN use appears to be acceptable among women in rural Uganda, setting the stage for further SmartNet field testing.

## 1. Introduction 

Approximately 3.4 billion people live at risk of malaria infection throughout the world and most of the more than 600,000 annual malaria-related deaths occur among Sub-Saharan African children under five years of age [1]. The United Nations Development Goals target a 75% reduction in malaria by 2015 [2]. Insecticide-treated bednets (ITNs) are a cost-effective means of malaria prevention [1, 3]. Ownership of an ITN reduces mortality by 18–23% [4], saving 5.5 lives per year for every 1000 ITNs distributed [5]. In 2013, the World Health Organization (WHO) recommended that free bednets be made available universally for anyone at risk of malaria [6].

Despite the acceptance of bednets as effective tools for malaria prevention, there are still significant questions about the factors that influence household decisions to use bednets [7]. Most studies assessing the determinants of bednet use rely on self-reports from households and individuals [8–12]. However, self-reported use may be prone to both social desirability and recall biases [13], which compromise both our understanding of barriers to bednet use and estimates of the true cost-effectiveness of bednets as a malaria prevention tool. The effects of these biases due to self-reporting on estimates of bednet use are not well defined in the literature, as there are very few studies which explicitly discuss the discrepancy between self-reports and more objective measures [14–16].

Compared to asking individuals or household representatives about their bednet use, more objective means of measuring bednet use have been attempted but are problematic. Spot checks during sleeping hours [17–19], while the most accurate, are invasive and unlikely to be acceptable to most populations. Visually confirming that bednets are mounted above sleeping areas in households [20–24] is also invasive, requiring entry into households, and does not ensure that a bednet is actually being unfurled at night.

**Figure 1:**
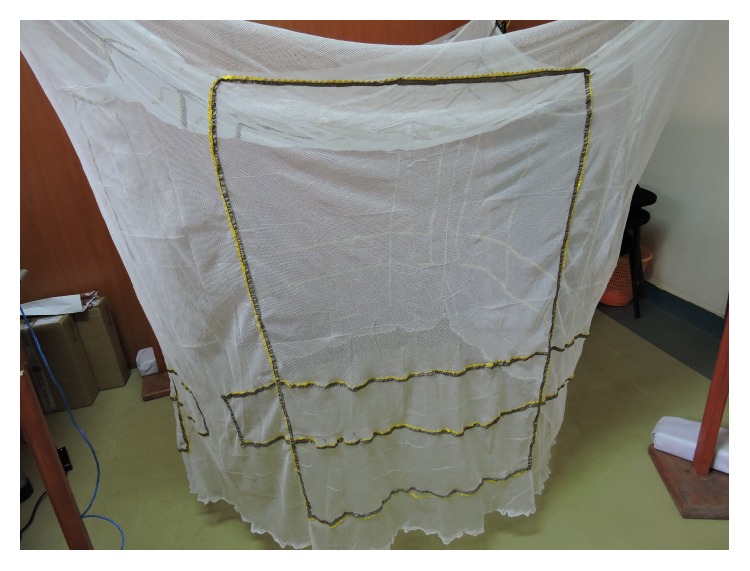
Side view of SmartNet.

**Figure 2:**
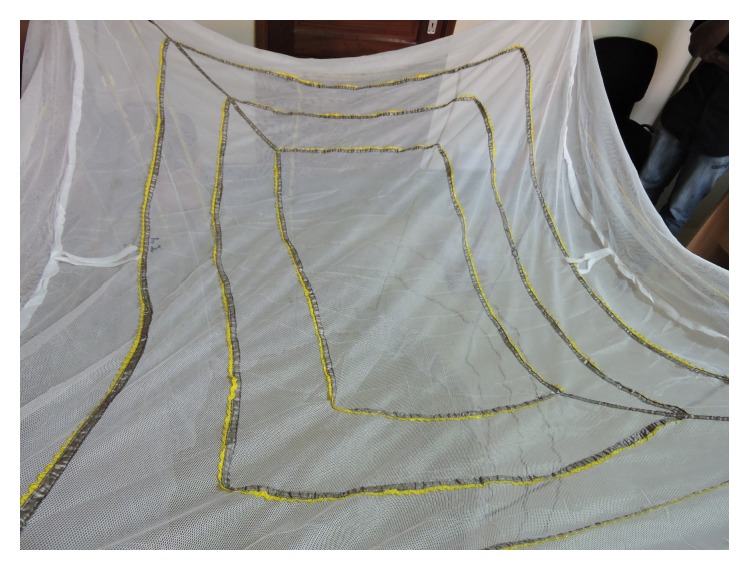
Top view of SmartNet.

**Figure 3:**
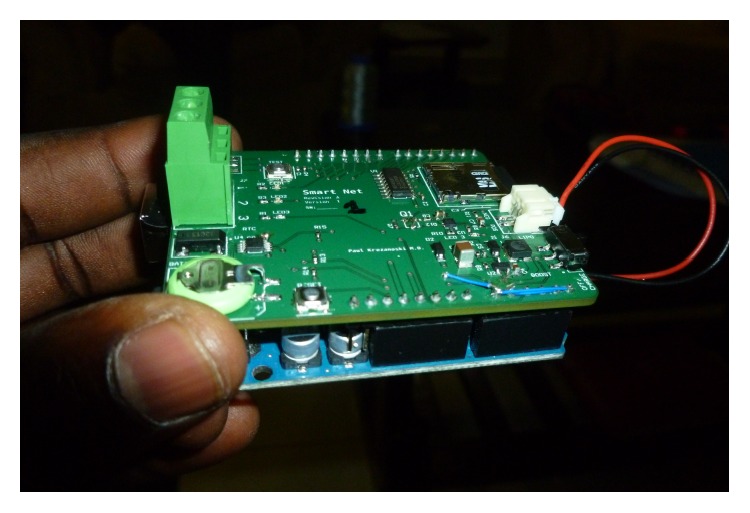
SmartNet microprocessor.

Real-time logging of bednet use for later analysis offers the possibility for more precise understanding of bednet use behaviors, in terms of both accuracy and temporal resolution, with less invasion than visual observation of use [25]. Furthermore, objective bednet use monitoring will be a key to understanding malaria epidemiology and prevention as mosquito biting behavior is known to change with bednet usage [26]. With these advantages of objective monitoring in mind, we have developed SmartNet, an electronic monitor of bednet use. SmartNet uses a microprocessor and conductive fabric sewn into a WHO-approved bednet in order to detect whether a bednet is unfurled or not. The unfurling of the bednet in a household is taken as a proxy of bednet use and, conversely, the folding up of the bednet is assumed to be a proxy of nonuse. The conductive material can be integrated into any size bednet. For this study, SmartNet components were integrated into white rectangular (5′ × 7′ × 7′) bednets bought from a local pharmacy (~$12). The conductive fabric is sewn to form three distinct electric circuits that make contact with each other when the bednet is folded or tied up for storage. A microprocessor sends an electrical current every 15 minutes to check circuit connectivity, allowing the SmartNet to distinguish between a net in use or not. Time-stamped data is stored on a removable memory card for later analysis (Figures 1, 2, and 3).

SmartNet is being actively developed through a collaboration between engineers and health professionals at Massachusetts General Hospital in Boston, MA, USA, and the Mbarara University of Science and Technology (MUST) at the Consortium for Affordable Medical Technologies (CAMTech) innovation center in Uganda. Development of SmartNet exemplifies a cocreation model for development of health technologies in developing countries [27]. Cocreation engages the end-users of technologies in developing countries to become innovators themselves through top-to-bottom involvement across the product development lifecycle.

It is envisioned that SmartNet could be utilized as a research tool for obtaining reliable measures of longitudinal bednet use in target populations or as a sampling tool to validate other forms of assessing bednet use, that is, self-reports. As a first step to understand the feasibility and acceptability of electronic bednet use monitoring, we examined the attitudes about SmartNet among mothers of young children and among pregnant women in malaria-endemic rural Uganda. After introducing the women to a model SmartNet and explaining its functionality, we conducted quantitative and semistructured qualitative interviews focused on impressions of SmartNet and attitudes about objective bednet monitoring.

## 2. Methods 

This study was conducted in southwestern Uganda at the Kinoni Health Center IV in Rugando Subcounty, Mbarara District. The health center, located 5 kilometers from Mbarara Town, serves about 110 patients per day, most of whom are rural subsistence farmers. Malaria is endemic to the region and makes up an estimated 20% of outpatient visits (16–25 visits/day) to the Kinoni Health Center.

We recruited 50 participants from July to August 2014. The number of participants was based on an attempt to capture the range of experiences with bednets and attitudes about SmartNet and electronic bednet monitoring. Participants were identified among patients waiting to see health providers at the clinic. They were invited to participate in the study after their clinic visit was complete. A room was set aside for confidential study visits. Informed consent was gathered from all participants before proceeding to the study interviews. Since our study focused on those at most risk of malaria, we recruited women to participate in the study if they were mothers of at least one child aged five years or younger or were currently pregnant. These recruitment criteria also allowed for selection of a study sample which is familiar with bednets, as our previous work suggests that households with young children are more likely to own and use bednets [28]. Participants were informed that their participation in this study would not affect future inclusion in SmartNet-related studies. No incentive was provided for participation. All consents and surveys were translated into Runyankole, the local language. All participants signed a written informed consent to participate. Participants were given the option to complete the survey in either English or Runyankole. In order to maintain consistency of interpretation of qualitative responses, a single research assistant (Nuriat Nambogo), who is fluent in Runyankole and English, conducted all interviews.

Participants received a structured interview to collect data on age, marital status, number of children, pregnancy status, education and literacy, employment, and average monthly income. Then they answered a questionnaire relating to their prior experience with antimalarial bednets, current use of bednets, frequency of household bednet use, and their opinions about the ease of use of bednets. Our prior work suggests that knowledge about malaria is a significant indicator of bednet use within a household [29]. All participants were shown a SmartNet, which was hanging in the interview room, and its components and functionality were described before participants were asked SmartNet-specific qualitative questions.

Participants answered open-ended questions about their impressions of SmartNet, their “likes” and “dislikes” about the device, individual concerns, and anticipated concerns about the device from the viewpoint of others within their household. This final line of questioning, from the viewpoint of others in the household, provided a level of abstraction in order for participants to provide honest comments without violating social norms. The responses were written on the survey sheet for later translation and coding. Themes were derived from a sample of qualitative responses to each question, and then each survey was coded independently by two members of the research team (Jeffrey I. Campbell and Nuriat Nambogo). Researchers discussed inconsistencies in coding, and when inconsistencies existed, a final code was established by consensus between the two coders or the primary author if consensus could not be reached.

All data were collected on paper forms and entered manually into the Research Electronic Data Capture (REDCap) tool hosted at Massachusetts General Hospital, Boston, MA, USA [30]. The data were cleaned following the completion of data entry. Quantitative data were analyzed in Stata 10 (Statacorp, College Rd, TX) and qualitative responses were reviewed and coded in Microsoft Excel.

## 3. Results

### 3.1. Demographic Characteristics

A total of 50 women were approached and all consented to participate. Participants had a mean age of 27 years and a mean of 2.3 children. Ten percent of the women had no children (5/50) and 8% (4/50) had more than two children under five years. Overall, twenty-two percent (11/50) of women reported being currently pregnant.

Fifty-four percent (27/50) of women had attended only primary school and 8% (4/50) had never attended school. Seventy-two percent (36/50) of women self-identified as farmers. The average self-reported monthly income was 162,250 Ugandan Shillings (UGX) (~$62.28) (range: 5,000 UGX to 1,500,000 UGX) (Table 1).

### 3.2. Bednet Ownership and Use

Ninety percent (45/50) of women reported that their household currently owned at least one bednet and 80% (40/50) reported that at least one bednet in their household was in use the night before the survey. Seventy-six percent (38/50) of women reported that a bednet was in use every day in the previous week by at least someone in their household. Eighty-four percent (42/50) reported that their household's ability to use bednets as directed was good or very good and 8% (4/50) reported their household's ability as poor or very poor. The households owned an average of 2.42 bednets (range: 1 to 6) (Table 2).

Self-reported bednet use was high, with 96% (48/50) of respondents reporting that they had used a bednet at some point in the past and 66% (33/50) reporting using a bednet every night in the previous week. Twenty percent (10/50) did not use a net at all in the last week and 14% (7/50) had not used a net in the last month. Among women who had ever used bednets, greater than 80% reported that bednets were easy or very easy to use and easy or very easy to remember to use. However, 14.6% (7/48) of ever-users reported that they were difficult to use and 10.4% (5/48) reported that they were difficult to remember to use. Reasons for not using bednets included excessive heat while under the net, traveling away from home or not owning enough nets.

### 3.3. Impressions of the SmartNet Device

All participants (50/50) said they would be interested in using the SmartNet in their homes. In open-ended responses, reasons that women gave included liking the appearance of the SmartNet, thinking the net would be useful and wanting to have a new net. Ninety-two percent (46/50) reported that they thought the device would be easy to use (Table 3). Notably, many of the responses to the question “are there particular things you like about the SmartNet?” pertained to nets in general and were independent of monitoring. For instance, many participants reported that they would like new nets because their current nets were old or had holes in them.

When queried about how their behavior would change in response to using SmartNet, 32% (16/50) of women reported that the SmartNet would not change their net use. No participants stated that their net use would decrease. Of the 68% (34/50) who reported an anticipated increase in net use, reasons included the fact of being monitored, being reminded by the device to use the net, and the attractive appearance of the net.

While the majority of responses to the SmartNet demonstration were positive, some participants offered suggestions or negative impressions of the net. Themes included fear of the device, perceived dislike of being studied when use of bed net is not as directed and dislike of home-visits to check on the net.

### 3.4. Perceptions of Objective Monitoring of Bednet Use

The majority of participants did not express personal concerns with objective monitoring. When participants were asked “how would you feel knowing that your bednet use is being monitored by the SmartNet?” ninety percent (45/50) of participants reported that they did not believe their family members would be worried about being monitored by SmartNet (Table 4).

When they did mention concerns, participants focused on monitoring of behaviors, including sleeping behaviors, while in bed at night:

“I would think that the device is recording me while in bed with my husband.”

“I would be thinking every time they are looking at me and measuring me while in my bed.”

More participants described how other people might be concerned with monitoring compared to those who reported being concerned themselves. Some other concerns focused around fear of detecting net misuse:

“Some people may not like to be monitored, especially those that at times do not use their nets.”

“For people who get discomfort with heat at night and they remove their nets, they may fear to do this because of being monitored.”

Several participants reported that monitoring would help them to improve their net usage and said they would appreciate feedback that the net could provide. For instance, one participant noted that the desire to “get good results” would help her to improve her net use. A few participants also mentioned the perceived benefits of participating in a research study involving SmartNet, including frequent interaction with study staff.

## 4. Discussion

In this mixed-method survey, mothers and pregnant women in rural Uganda, who were well acquainted with bednets, expressed favorable views regarding using SmartNet for objective monitoring of their households' bednet use. While a minority of participants expressed concern about being monitored at night while they slept, all participants were nevertheless willing to use a SmartNet in their home.

SmartNet was developed out of a concern that self-reported use of bednets is inaccurate and temporally imprecise. Most studies of bednet use rely on self-reported use. While some studies have relied on more objective measurements to reduce bias, the majority of these have been one-time assessments with visual observation of hanging bednets or individuals under bednets. Since effective malaria prevention requires consistent correct bednet use to protect against mosquitoes every night, one-time surveys of use are unable to capture a temporally complete adherence record. The ideal adherence monitor for bednet use must capture nightly adherence data. We are aware of only one other study that has used an objective, nightly adherence tool [21]. This study found that reports of use the night before correlated well with objective use data from a motion detector. However, there were significant discrepancies in reports about net use a week prior and 2–4 weeks prior, confirming the presence of recall bias. This study did not report on the views of participants about the monitoring of their bednet use.

While multiple studies have used qualitative methods to examine perceptions of malaria and bednet use throughout Sub-Saharan Africa [31, 32], we are unaware of any study which has made as its primary aim the exploration of study subjects' attitudes towards objective monitoring of bednet use. Perceptions of the monitoring itself are particularly important when developing tools for electronic adherence monitoring, in order to understand how a tool will be used and to ensure that monitoring is culturally acceptable.

An important question is whether the monitoring itself will change behaviors. This is the well-known “Hawthorne effect,” whereby individuals modify their behavior merely as a result of being monitored. For instance, women in the study reported that they thought the use of SmartNet would improve their regular use of bednets in a desire to “get good results.” Determining how objective bednet monitoring influences bednet behavior is an important area of future research.

Data gathered in our study highlighted several areas for design modifications for SmartNet and for similar objective bednet monitors. In particular, participants reported a fear that the uncovered logging device for capturing use data could be damaged. Future improvements could include better protection and concealment of the logging device. In addition, some participants reported a dislike of household visits to gather net use data. Improvements in battery life or, potentially, wireless transmission of data, could mitigate this concern. Predeployment studies such as this one are crucial components of a cocreation ethic [22], whereby real-time data about user impressions of technologies are fed back into design improvements. Further refinements of the SmartNet device are already underway in Uganda at the CAMTech Innovation laboratory (http://www.massgeneralcenterforglobalhealth.org/camtech/) offering an enhanced responsiveness to local views of the SmartNet technology.

There are several limitations of this study. The participants did not actually use the SmartNet, so these results are perceptions of participants based on theoretical use. Further design improvement and perceptions data will come from acceptability components of longer field studies among households using SmartNet devices. Because of the focus on bednet use to prevent maternal and child malaria, men were not interviewed and may hold different views of bednets, objective monitoring of bednet use, and SmartNet in particular. Future studies will explore this in more detail. The responses to this predeployment attitudes survey could be prone to social desirability bias providing an overly positive impression of the device. Women were told before joining this study that their answers would make no difference in ultimately being chosen for use of a SmartNet in future studies. Nevertheless, some of the perceptions of SmartNet suggest that women indicated interest in SmartNet as a means of obtaining a free bednet, perhaps inflating the apparent acceptability of the technology. The study participants were drawn from a convenience sample of women visiting one health center in the community of interest and therefore the study results may suffer from selection bias related to this choice. Finally, SmartNet measures whether or not a bednet is unfurled but does not measure who is under the bednet or whether the bednet is in perfect use.

## 5. Conclusion

Objective monitoring of ITN use appears to be acceptable among women in rural Uganda. Concerns about monitoring focused on the appearance of the device and the idea of being monitored while sleeping. These results will inform SmartNet device improvements and set the stage for future field-based feasibility testing.

## Figures and Tables

**Table 1:** Demographic characteristics.

Total number of respondents	*n* = 50
Age (mean ± SD)	27 ± 5.6
	Range: 17–40
Pregnant (*n* [%])	11 (22%)
Gestational months of pregnancy (*n* = 11) (mean ± SD)	5.7 ± 2.1
	Range: 3–9
Number of children under 5 years of age (mean ± SD)	2.8 ± 1.6
	Range: 0–8
Number of children under five years (*n* [%])	
0	5 (10%)
1	22 (44%)
2	19 (38%)
3	4 (8%)
Highest education attained (*n* [%])	
Attended up to	
Primary school	27 (54%)
Secondary school	17 (34%)
Tertiary schooling	2 (4%)
Never attended school	4 (8%)
Literacy (*n* [%])	
Read sample text completely	31 (62%)
Read sample text partially	7 (14%)
Unable to read sample text	12 (24%)
Able to write name legibly (*n* [%])	
Yes	44 (88%)
No	6 (12%)
Occupation (*n* [%])	
Farmer	36 (72%)
Salaried employee	5 (10%)
Own shop	3 (6%)
Other casual labor	6 (12%)
Average monthly income (mean ± SD)	162,250 UGX
	Range: 5,000–1,500,000

**Table 2:** Bednet ownership and use.

*Household bednet characteristics*	*n* = 50
Household bednet ownership (*n* [%])	45 (90%)
Average number of bednets per household (mean ± SD)	2.42 ± 1.12
	Range: 1–6
Household bednet use the night before (*n* [%])	40 (80%)
Household net use by someone in the week before (days) (*n* [%])	
7	38 (76%)
<7	6 (12%)
0	5 (10%)
No response	1 (2%)
Household's ability to use bednets correctly (*n* [%])	
Very good	19 (38%)
Good	23 (46%)
Fair	3 (6%)
Poor	2 (4%)
Very poor	2 (4%)
No response	1 (2%)
*Individual bednet characteristics*	
Ever used bednet (*n* [%])	48 (96%)
Personally used every night last week (*n* [%])	33 (66%)
Did not use in last week (*n* [%])	10 (20%)
Did not use in last month (*n* [%])	14 (28%)
Bednets are (*n* [%])	
Very easy to use	27 (54%)
Easy to use	13 (26%)
Neither easy nor hard to use	2 (4%)
Difficult to use	6 (12%)
Very difficult to use	1 (2%)
No response	1 (2%)
Remembering to use a bednet is (*n* [%])	
Very easy	30 (60%)
Easy	13 (26%)
Neither easy nor hard	1 (2%)
Difficult	5 (10%)
Very difficult	0 (0%)
No response	1 (2%)
Agree that bednets are useful (*n* [%])	48 (96%)
*Reasons for not using a net* (*n* = 25) (*n* [%])	
Too hot	8 (16%)
Traveled away from home	6 (12%)
Not enough nets	6 (12%)
Allergic/dislike treatment	4 (8%)
Net damaged or being washed	2 (4%)
Spouse does not want to use it	1 (2%)

**Table 3:** Impressions of SmartNet device.

Interested in using (*n* [%])	50 (100%)
Perceived to be easy to use (*n* [%])	46 (92%)

Positive impressions	
Theme	Example

Looks/feels nice or attractive	“I love the yellow color and the designs on it”
Looks easy to use	“I can hang it well on my walls, because it looks easy to hang. It is also light enough to move with”
Would increase usage	“I would be more accurate in putting [up] my net”
Would help to learn how to use nets	“I want the researchers to monitor me and know if I use my net well or not, which can help me improve in case I don't use it well”
Help with malaria prevention in the community	“If these nets are given out it would reduce on the malaria cases especially in villages”
Help learn about net use	“They [researchers] will be able to find out how best people can use their nets”

Negative impressions	
Theme	Example

Appearance	“Some people may not like this color of the net since they have children who can dirty it”
Treatment	“If it has a lot of chemicals it will disturb me”
Net would be difficult to hang	“The squared net might be difficult to some people in hanging”
Device could be damaged	“In case the device is not covered, and it stays open, people may spoil it especially children”
People who misuse nets may not like the SmartNet	“For those who don't use the nets, they may not like it when you come to check on their bednets as you monitor them”
The device could negatively affect health	“They may fear that it [SmartNet] might negatively affect their health”
Dislike of home visits	“Some people who are not hygienically good in their bedrooms may not like you entering their bedrooms”
Not able to wash the net	“They may fear washing the net if it is to be washed”
Fear that the device may be stolen	“Fearing that thieves may steal the device from them”

**Table 4:** Impressions of objective monitoring of bednet use.

Personally not worried about monitoring (*n* [%])	45 (90%)
Family would not be worried about monitoring (*n* [%])	43 (86%)

Positive impressions	
Theme	Example

Monitoring would improve use	“Because I would want to get good results about the use”
Provides feedback	“If I can be given feedback on how I use my net, it can help me improve in case I don't use it well”
Benefits of participating in a research study	“It would mean that the researchers always will remember to check on me and visit me at home”

Negative impressions	
Theme	Example

Monitoring while sleeping	“People might think that they device will be monitoring them as well when they are sleeping in their beds under the net”
Monitoring of misuse	“Some people may not like to be monitored, especially those that at times do not use their nets”
Change current behaviors	“For people who get discomfort with heat at night and they remove their nets, they may fear to do this because of being monitored”
Concern about continuous monitoring	“I would be thinking every time they are looking at me and measuring me while in my bed”
Concern about recording private behavior	“I would think that the device is recording me while in bed with my husband”

## References

[1] World Health Organization (2013). *World Malaria Report*.

[2] United Nations (2014). *Millenium Development Goals: Combat HIV/AIDS, Malaria and Other Diseases*.

[3] Flaxman A. D., Fullman N., Otten M. W. (2010). Rapid scaling up of insecticide-treated bed net coverage in Africa and its relationship with development assistance for health: a systematic synthesis of supply, distribution, and household survey data. *PLoS Medicine*.

[4] Lim S. S., Fullman N., Stokes A. (2011). Net benefits: a multicountry analysis of observational data examining associations between insecticide-treated mosquito nets and health outcomes. *PLoS Medicine*.

[5] Lengeler C. (2004). Insecticide-treated bed nets and curtains for preventing malaria. *Cochrane Database Of Systematic Reviews*.

[6] World Health Organization (2013). *WHO Recommendations for Achieving Universal Coverage with Long-Lasting Insecticidal Nets for Malaria Control*.

[7] Pulford J., Hetzel M. W., Bryant M., Siba P. M., Mueller I. (2011). Reported reasons for not using a mosquito net when one is available: a review of the published literature. *Malaria Journal*.

[8] Belay M., Deressa W. (2008). Use of insecticide treated nets by pregnant women and associated factors in a pre-dominantly rural population in northern Ethiopia. *Tropical Medicine & International Health*.

[9] Fernando S. D., Abeyasinghe R. R., Galappaththy G. N. L., Gunawardena N., Ranasinghe A. C. R., Rajapaksa L. C. (2009). Sleeping arrangements under long-lasting impregnated mosquito nets: differences during low and high malaria transmission seasons. *Transactions of the Royal Society of Tropical Medicine and Hygiene*.

[10] Skarbinski J., Massaga J. J., Rowe A. K., Kachur S. P. (2007). Distribution of free untreated bednets bundled with insecticide via an integrated child health campaign in Lindi Region, Tanzania: lessons for future campaigns. *American Journal of Tropical Medicine and Hygiene*.

[11] Kulkarni M. A., Vanden Eng J., Desrochers R. E. (2010). Contribution of integrated campaign distribution of long-lasting insecticidal nets to coverage of target groups and total populations in malaria-endemic areas in Madagascar. *American Journal of Tropical Medicine and Hygiene*.

[12] Becker-Dreps S. I., Biddle A. K., Pettifor A. (2009). Cost-effectiveness of adding bed net distribution for malaria prevention to antenatal services in Kinshasa, Democratic Republic of the Congo. *The American Journal of Tropical Medicine and Hygiene*.

[13] Yohannes K., Dulhunty J. M., Kourleoutov C. (2000). Malaria control in central Malaita, Solomon Islands. 1. The use of insecticide-impregnated bed nets. *Acta Tropica*.

[14] Skarbinski J., Winston C. A., Massaga J. J., Kachur S. P., Rowe A. K. (2008). Assessing the validity of health facility-based data on insecticide-treated bednet possession and use: comparison of data collected via health facility and household surveys—Lindi region and Rufiji district, Tanzania, 2005. *Tropical Medicine and International Health*.

[15] Korenromp E. L., Miller J., Cibulskis R. E., Cham M. K., Alnwick D., Dye C. (2003). Monitoring mosquito net coverage for malaria control in Africa: possession vs. use by children under 5 years. *Tropical Medicine and International Health*.

[16] Wong J., Shah M. P., Mwandama D., Gimnig J. E., Lindblade K. A., Mathanga D. P. (2015). Home visits to assess the reliability of caregiver-reported use of insecticide-treated bednets by children in Machinga District, Malawi. *American Journal of Tropical Medicine and Hygiene*.

[17] Iwashita H., Dida G., Futami K. (2010). Sleeping arrangement and house structure affect bed net use in villages along Lake Victoria. *Malaria Journal*.

[18] Alaii J. A., Hawley W. A., Kolczak M. S. (2003). Factors affecting use of permethrin-treated bed nets during a randomized controlled trial in Western Kenya. *The American Journal of Tropical Medicine and Hygiene*.

[19] Frey C., Traoré C., De Allegri M., Kouyaté B., Müller O. (2006). Compliance of young children with ITN protection in rural Burkina Faso. *Malaria Journal*.

[20] Krezanoski P. J., Comfort A. B., Hamer D. H. (2010). Effect of incentives on insecticide-treated bed net use in sub-Saharan Africa: a cluster randomized trial in Madagascar. *Malaria Journal*.

[21] Fink G., Masiye F. (2012). Assessing the impact of scaling-up bednet coverage through agricultural loan programmes: evidence from a cluster randomised controlled trial in Katete, Zambia. *Transactions of the Royal Society of Tropical Medicine and Hygiene*.

[22] Spencer S., Grant A. D., Piola P. (2004). Malaria in camps for internally-displaced persons in Uganda: evaluation of an insecticide-treated bednet distribution programme. *Transactions of the Royal Society of Tropical Medicine and Hygiene*.

[23] Gerstl S., Dunkley S., Mukhtar A. (2010). Long-lasting insecticide-treated net usage in eastern Sierra Leone—the success of free distribution. *Tropical Medicine & International Health*.

[24] Wolkon A., Vanden Eng J. L., Morgah K. (2010). Rapid scale-up of long-lasting insecticide-treated bed nets through integration into the national immunization program during child health week in Togo, 2004. *The American Journal of Tropical Medicine and Hygiene*.

[25] Koudou B. G., Malone D., Hemingway J. (2014). The use of motion detectors to estimate net usage by householders, in relation to mosquito density in central Cote d'Ivoire: preliminary results. *Parasites and Vectors*.

[26] Russell T. L., Govella N. J., Azizi S., Drakeley C. J., Kachur S. P., Killeen G. F. (2011). Increased proportions of outdoor feeding among residual malaria vector populations following increased use of insecticide-treated nets in rural Tanzania. *Malaria Journal*.

[27] Caldwell A., Young A., Gomez-Marquez J., Olson K. R. (2011). Global health technology 2.0. *IEEE Pulse*.

[28] Krezanoski P. J., Comfort A. B., Tsai A. C., Bangsberg D. R. (2014). Households with young children and use of freely distributed bednets in rural Madagascar. *International Health*.

[29] Krezanoski P. J., Tsai A. C., Hamer D. H., Comfort A. B., Bangsberg D. R. (2014). Household malaria knowledge and its association with bednet ownership in settings without large-scale distribution programs: evidence from rural Madagascar. *Journal of Global Health*.

[30] Harris P. A., Taylor R., Thielke R., Payne J., Gonzalez N., Conde J. G. (2009). Research electronic data capture (REDCap)-a metadata-driven methodology and workflow process for providing translational research informatics support. *Journal of Biomedical Informatics*.

[31] Kachur S. P., Phillips-Howard P. A., Odhacha A. M., Ruebush T. K., Oloo A. J., Nahlen B. L. (1999). Maintenance and sustained use of insecticide-treated bednets and curtains three years after a controlled trial in western Kenya. *Tropical Medicine & International Health*.

[32] Toé L. P., Skovmand O., Dabiré K. R. (2009). Decreased motivation in the use of insecticide-treated nets in a malaria endemic area in Burkina Faso. *Malaria Journal*.

